# Investigating porcine parvoviruses genogroup 2 infection using in situ polymerase chain reaction

**DOI:** 10.1186/s12917-018-1487-z

**Published:** 2018-05-21

**Authors:** Dinko Novosel, Daniel Cadar, Tamás Tuboly, Andreja Jungic, Tomasz Stadejek, Tahar Ait-Ali, Attila Cságola

**Affiliations:** 1 Department of Pathology, Croatian Veterinary Institute, Savska cesta 143, 10000 Zagreb, Croatia; 2 Department for Animal science, Faculty of Agriculture, University of Zagreb, Svetosimunska 25, 10000 Zagreb, Croatia; 3 WHO Collaborating Centre for Arbovirus and Haemorrhagic Fever Reference and Research, National Reference Centre for Tropical Infectious Diseases, Bernhard Nocht Institute for Tropical Medicine, Bernhard-Nocht-Strasse 74, 20359 Hamburg, Germany; 4 Department of Microbiology and Infectious Diseases, Faculty of Veterinary Science, Immunology, Szent István University, István u. 2, Budapest, 1078 Hungary; 5 Department for Virology, Croatian Veterinary Institute, Savska cesta 143, 10000 Zagreb, Croatia; 6 Department of Pathology and Veterinary Diagnostic, Faculty of Veterinary Medicine, University of Life Science, Nowoursynowska 159C, 02-776 Warsaw, Poland; 7 The Roslin Institute and Royal (Dick) School of Veterinary Studies, University of Edinburgh, Easter Bush, Edingburgh, United Kingdom

**Keywords:** In situ PCR, PPV2, Lungs, Lymphocytes

## Abstract

**Background:**

Porcine parvovirus 2 (PPV2) was detected in swine serum without showing any relationship with disease. The emergence of the virus seemed to be a unique event until other genetically highly similar parvoviruses were identified in China and, later in 2012, the presence of the virus was also described in Europe. PPV2 is widely distributed in pig populations where it is suspected to be involved in respiratory conditions, based on its frequent detection in lung samples. In order to investigate the potential pathogenic involvement of PPV2, 60 dead pigs were examined from two farms. They were necropsied and tested for PPV2 and PCV2 (Porcine circovirus type 2) by PCR; by Brown and Brenn (B&B) staining for bacteria; by immunohistochemistry (IHC) to detect CD3, Swine leukocyte antigen class II DQ (SLAIIDQ), lysozyme, porcine reproductive and respiratory syndrome virus (PRRSV), swine influenza (SIV), *Mycoplasma hyopneumoniae (Mhyo)*; and by in situ hybridization (ISH) to detect ssDNA and dsDNA of PCV2. PPV2 positive samples were subjected to in situ polymerase chain reaction (IS-PCR) including double staining method to detect PPV2 and host cell markers. To calculate statistical difference we used GENMOD or LOGISTIC procedures in Statistical Analysis System (SAS®).

**Results:**

We found that the PPV2 was localized mostly in lymphocytes in lungs, lymph nodes and liver. Neither CD3 antigen nor lysozyme was expressed by these infected cells. In contrast, low levels of SLAIIDQ were expressed by infected cells, suggesting that PPV2 may have a specific tropism for immature B lymphocytes and/or NK lymphocytes though possibly not T lymphocytes.

**Conclusion:**

The overall conclusion of this study indicates that PPV2 may contribute to the pathogenesis of pneumonia.

**Electronic supplementary material:**

The online version of this article (10.1186/s12917-018-1487-z) contains supplementary material, which is available to authorized users.

## Background

Parvoviruses are small, non-enveloped icosahedral viruses that are ubiquitous in animal species. The *Parvoviridae* family is composed of two subfamilies: *Parvovirinae*, which infect vertebrates, and *Densovirinae*. *Parvovirinae* can be further divided into eight genera [1]. A number of parvoviruses (PPV) that infect pigs have been identified and include the classical PPV type 1 (PPV1, recently grouped into the *Protoparvovirus* genus as Ungulate protoparvovirus 1), PPV2 belonging to the *Tetraparvovirus* genus (*Ungulate tetraparvovirus 3*) and PPV3 (also known as *Hokovirus*), belong to the *Tetraparvovirus* genus (*Ungulate tetraparvovirus* 2), whereas PPV4 is a representative of the *Copiparvovirus* genus (*Ungulate copiparvovirus 2*). Two additional PPVs, namely PPV5 [2] and PPV6 [3], are still unclassified *Protoparvovirus*). Porcine bocaviruses are also prevalent in pigs and are members of the *Bocaparvovirus* genus, *Parvovirinae* subfamily [4]. All of these viruses are genetically divergent from each other [5–12]. Genomes of PPVs are single-stranded DNA molecules, with a size of approximately 4–6.3 kilobases (kb), that carry terminal palindromic sequences [5]. In general, they possess two major open reading frames (ORFs) at e 5′- and ‘-3 end which encode for non-structural and capsid protein(s), respectively. An additional ORF3 exists in the middle of the viral genome in members of the *Copiparvovirus*, *Tetraparvovirus Bocaparvovirus* genus [5, 13]. The first newly discovered PPV species was PPV2 in 2005, which was detected in serum of otherwise clinically normal pigs [12]. The emergence of the virus appeared to be a unique event until befor 2006/7, when other genetically similar parvoviruses were identified in China [14], where PPV2 sequences were detected in serum from pigs with marked pyrexia and PCV2-induced post-weaning multi-systemic wasting syndrome (PMWS). Later, in 2012, PPV2 was also described in Europe [15]. Phylodynamic analysis indicated that PPV2 had likely been present in Europe since 1920 in domestic and sylvatic hosts, representing a possible source of the virus [16]. Nowdays PPV2 is considered worldwide distributed in pig populations [15, 17, 18] and according to the frequent detection of the virus in lung samples it is suspected to be involved in respiratory conditions of pigs [2, 6, 19]. Recent investigations have indicated that PPV2 infection was correlated with the outbreak of clinically overt respiratory disease in pigs [20]. The aim of this study was to detect PPV2 using IS-PCR in order to investigate its possible implication in pathology of the lung and evaluate its association with PCV2 infections. In addition, we also applied immunohistochemistry to determine expression of host immune markers in infected cells.

## Results

**Fig. 1:**
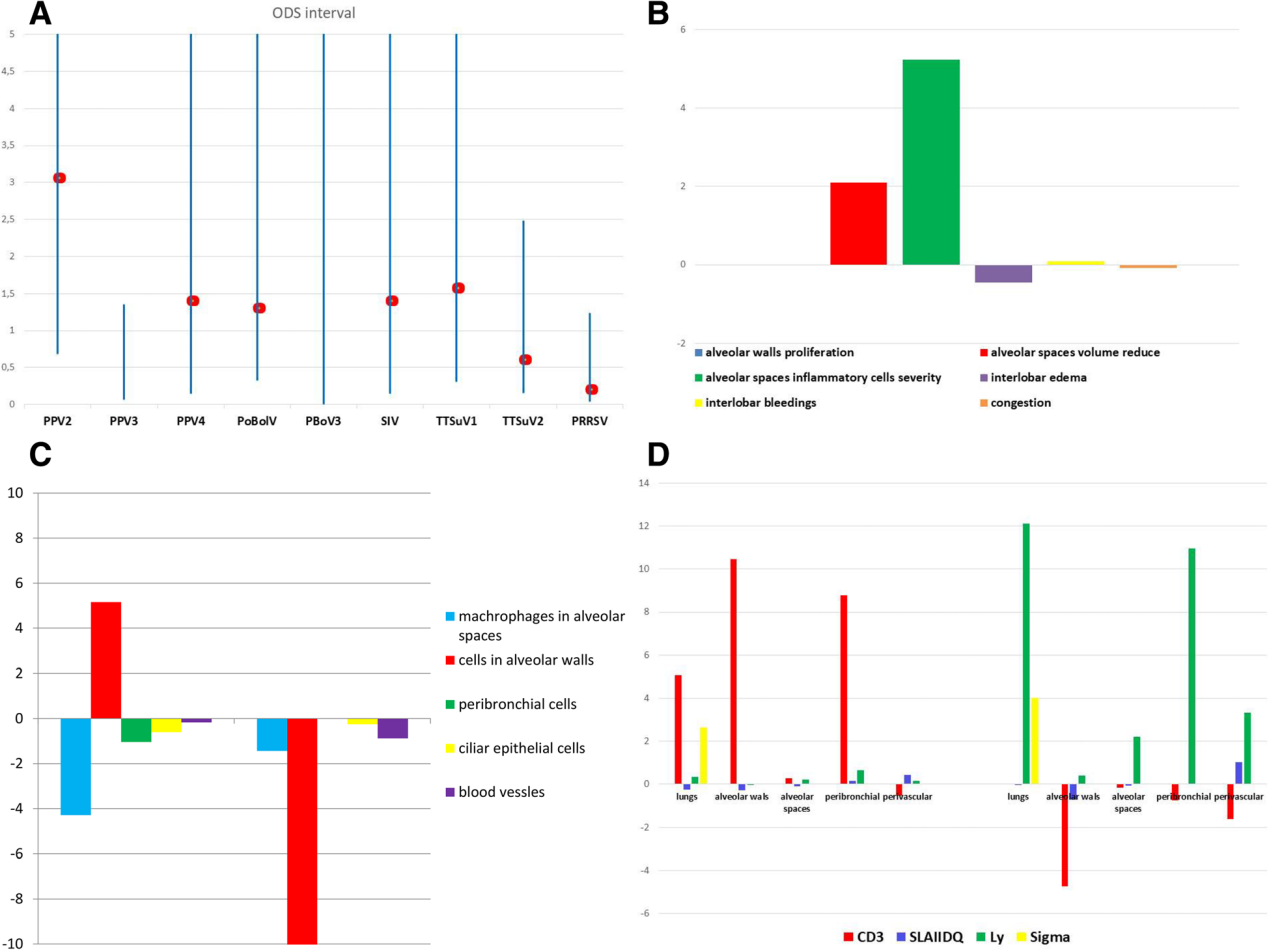
ODDS ratio **a** for detected virus and PMWS; Wald Chi^2^ test to evaluate the statistical significance of **b** specific lesions observed in lungs; **c** amount and level of replication PCV2 in PPV2 positive and PPV2 negative animals. **d** statistical significance in difference of presence of T lymphocytes, B lymphocytes and macrophages in alveolar walls; in alveolar spaces; in peribronchial area and in perivascular area in PPV2 positive and PPV2 negative. Positive Wald Chi2 values means that observed lesion was more frequently observed in PPV2 positive animals while negative Wald CHi2 value means that observed lesion was less frequently observed in PPV2 negative animals

**Fig. 2:**
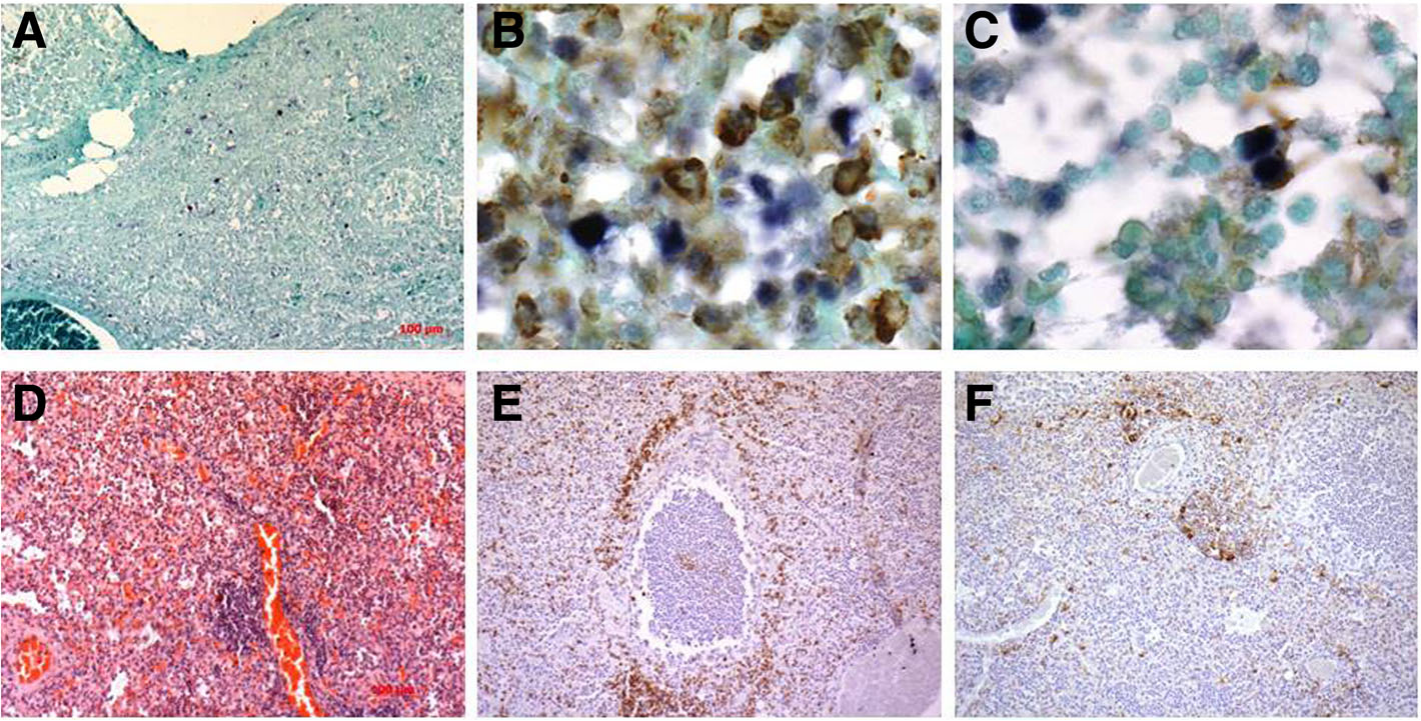
Lungs T3. **a** Double staining IHC to detect CD3, DAB chromogen/IS PCR to detect PPV2, NBT chromogen 40X; **b** Double staining IHC to detect SLAIIDQ, DAB chromogen/IS PCR to detect PPV2, NBT chromogen 40X; **c** IS PCR to detect PPV2, NBT chromogen, 10X. **d** Severe pneumonia, H&E, 5X**, e** IHC to detect CD3, DAB chromogen, counterstained with Hematoxyline, 5X, **f** IHC to detect SLAIIDQ, DAB chromogen, counterstained with Hematoxyline 5X

Of the 47 lungs tested, 24 were positive for the presence of PPV2 by PCR in farm E. All samples were positive for PCV2 by PCR (Additional file 1). However, using ISH only, 3 and 6 samples were weak and negative for PCV2, respectively, while the rest ranged from moderately to strongly positive (Additional file 1). Using PCR, several other viruses were confirmed in lungs including PPV3, PPV4, TTSuV1, TTSuV2, PoBolV, PoBoV3. PRRSV and SIV, while lungs were negative for presence of PPV1, PoBo1, 2 and 4, ADV and 6 V/7 V. Only 6 and 5 samples out of 47 were positive for PRRSV and SIV by PCR, however they were negative by IHC, respectively (Additional file 1). Statistical significance of the contribution of each virus in outbreak of PMWS is shown in Fig. 1a (Additional file 2). All examined lungs from farm E had a wide range of histopathological lesions and different PCV2 loads (Additional files 3 and 4). The statistical significance of these data is provided in Fig. 1b-d (Additional files 5, 6 and 7). We observed that the presence of PPV2 infection in the lung was significantly associated with a wide range of lesions (Fig. 1b). Notably, PPV2 infection correlated with a reduction in the size of the alveolar spaces, as well as increased numbers of CD3 cells in lungs (Fig. 1d) and a statistically significant increase of PCV2 load in cells of alveolar walls associated with a reduced replication in same cells (Fig. 1c). The level, intensity and aggregation of T lymphocytes, B lymphocytes and macrophages in specific parts of lungs were evaluated and correlated with the presence of PPV2 using IHC (Additional file 8). PPV2 was associated with increased levels of T-lymphocytes in the alveolar spaces and peribronchial area (Fig. 1d). On the other hand, PMWS strongly influenced the level of macrophages. PPV2 was detected in the lungs of 4 of 13 animals from farm T. Results are summarised in Additional files 9 and 10. Using IS-PCR a moderate to severe PPV2 infection was detected in three lung samples (T2, T3 and T7) (Fig. 2a-c). Extensive areas exhibited positive signals were mostly found in cells morphologically characteristic of lymphocytes and macrophages in alveolar spaces (Fig. 2d). The presence of PPV2 in lung tissue was associated with marked vascular engorgement, lymphocytic infiltration, reduction of alveolar spaces, necrosis of ciliary epithelial cells and bronchial lumens filled with mucus and inflammatory cells (Fig. 2d). There was a moderate to severe infiltrate of T lymphocytes around bronchi whereas limited B lymphocytes were present around blood vessels (Fig. 2e). Macrophages were found throughout the lung sections, mostly in alveolar walls and spaces. PPV2 infected cells did not express any of the host markers (Fig. 2a-c) apart from SLAIIDQ antigen, expressed at a low level (Fig. 2f). All cases were negative for PCV2 by PCR and ISH. In contrast, every tested lung was positive for *Mhyo*, where a small amount of the surface lipo-protein was identified in macrophages (data not shown).

## Discussion

The aim of this research was to investigate the potential involvement of PPV2 in lung pathology and its contribution to the pathogenesis of PCV2 associated diseases using in situ methods. PPV2 infections were detected on farm E by PCR but IS-PCR failed to detect the virus in situ. In contrast, PPV2 was detected using IS-PCR in non-PMWS affected pigs from farm T. IS-PCR is rarely employed therefore the experimental conditions had to be optimized to reach optimal sensitivity and specificity. PCR cycling conditions were based upon a pre-optimized method previously used to detect PPV2 using classic PCR. As described in the Materials and Methods, reducing the formalin fixation, protease pre-treatments, PCR hot start and use of single primer controls help to substantially improve the sensitivity and specificity of IS-PCR by reducing nucleic acid fragmentation and mis-priming during PCR amplification [21–24]. It is likely that further improvements will be required to increase further the sensitivity of the detection and decrease background such as diffuse non-specific staining.

### Deciphering PPV2 tropism

Positive signals for PPV2 by IS-PCR were detected in lungs tissue in cells with morphology consistent with lymphocytes and macrophages. These results coincide with the expected nature of parvoviruses in general, such as their requirement for rapid cellular division as it is commonly seen during the S-phase of cell cycle [25, 26]. To further define the nature of the infected cells, double staining methods capable of simultaneously detecting either CD3, SLAIIDQ or lysozyme host markers and PPV2 were optimized. In the lung tissue, PPV2 infected cells were associated with cells poorly expressing SLAIIDQ antigen and, occasionally, CD3, but not lysozyme. Based on these observations it is tempting to suggest that PPV2 may have a tropism for SLAIIDQ-positive cells. It is noteworthy that, although CD3 antigen is exclusive to pro-thymocytes or mature T-cells, SLAIIDQ antigen can be expressed by B-cells, antigen presenting cells and subsets of resting and activated T-cells [27]. For example, porcine bone marrow progenitor cells have been shown to express low levels of SLA class II [28]. Furthermore, SLAIIDQ and CD3 antigens are not found on natural killer lymphocytes. Taken together, our investigations highlight a potential tropism of PPV2 for lymphocytes. However, because of the possibility that a virus may alter the regulation of SLAIIDQ or CD3 advocate the need for further investigations to ascertain the exact nature PPV2 infection.

### Involvement of PPV2 infection in the pathology of lungs

The results from farm E suggest that PPV2 viruses may be associated with certain histopathological lesions and host immune response alterations. All the samples on farm E were PCV2-positive and as a consequence may have been a confounding factor in this work. Nevertheless, our analysis indicates that PPV2 may have implication in the pathology of the lungs. Our statistical analysis indicates that the significance is higher if PMWS is observed. In lungs, irrespectively of the presence of PCV2, PPV2 was often associated with a severe reduction in size of alveolar spaces accompanied with an increase in the number of T lymphocytes in alveolar walls and peri-bronchial inflammatory reaction while significance is even higher when PMWS is observed. Whereas similar pathological conditions have often been attributed to infection by classical pathogens such as PCV2 [29], PRRSV [30] and SIV [31], in farm T none of the lung tissues tested positive for any of these classical viruses. Similarly, *Mycoplasma hyopneumonia* was not detected in areas where there was a strong peribronchial T-cell infiltrate [32]. Since none of these lesions could be directly attributed to any known pathogens and since there was a heavy PPV2 load in the lung parenchyma, associated with inflammatory lesions, we speculate that this virus may be a significant contributing factor in the development of those lesions. This conclusion is in agreement with the recent observation by Csagola and colleagues (2016) [20] which indicates that PPV2 infections are associated with respiratory health problems [20].

The results obtained in this study also indicate that IS PCR is a valuable diagnostic tool for the detection of PPV2.

## Conclusions

We observed that PPV2 was localized mostly in lymphocytes in the lungs. Neither CD3 antigen nor lysozyme was expressed by these infected cells. In contrast, a low level of SLAIIDQ was expressed by infected cells, suggesting that PPV2 may have a specific tropism for immature B lymphocytes and/or NK lymphocytes, rather than T lymphocytes. Furthermore, we conclude that PPV2 may contribute to the pathogenesis of pneumonia. Overall, these observations are consistent with the general nature of parvoviruses which enter into organism through the oropharyngeal route and replicate in lymphoid cells to ultimately alter the lymphoid system and the gastrointestinal tract. Similarly, canine minute virus (*Canine bocaparvovirus* 1) has been found to cause interstitial pneumonia and lymphoid necrosis in Peyer’s patches [33]. Therefore, it is possible that all the lesions we have reported may be attributable, at least in part, to porcine parvovirus infection. To gather further evidence for this hypothesis, infections of SPF pigs will be required once in vitro PPV2 production in cell cultures is resolved.

## Methods

### Samples

Lungs were collected from 47 dead weaned pigs from a PMWS-positive farm (Farm E) and from 13 two to three-month-old dead pigs from a PMWS-negative farm (Farm T), both in Hungary. After post-mortem examination lungs were fixed in 10% neutral buffered formalin for 5–6 h, routinely processed, paraffin wax embedded, sectioned and stained for light microscopy as described above. To determine the presence of bacteria in tissues Brown and Brenn (B&B) staining method was used [34]. Parallel fresh lung samples were collected and stored at − 20 °C until processing. Lungs were tested for PPV2 and PCV2 by polymerase chain reaction (PCR). PPV2 complete genome sequence was revealed, designated as strain PPV2_Ted and deposited in Genbank, (access number: KX517759). Besides viruses of interest for this study all samples were checked for presence of Porcine parvovirus 1, 3 and 4, porcine Boca like virus (PBlV), Porcine boca virus 1, 2, 3 and 4 (PBoV), Torque teno sus virus 1 and 2 (TTSuV), Porcine cytomegalovirus (PCMV) (only in farm T), Aujezsky disease virus (ADV), Porcine reproductive and respiratory syndrome virus (PRRSV), Swine influence virus (H1N1/H3N2) (SIV) and Swine adenovirus (6 V/7 V). Paraffin wax embedded tissue was subjected to immunohistochemistry (IHC) to detect CD3, swine leukocyte antigen class II DQ (SLAIIDQ), lysozyme, PRRSV, swine influenza (SIV), *Mycoplasma hyopneumoniae (Mhyo)*, and ISH to detect ssDNA and dsDNA of PCV2. PPV2 positive samples were subjected to in situ PCR to detect the virus.

### PCR test for PCV2,TTSuV, PPV1–4, PoBoV1–4, PoBolV, 6 V/7 V, PCMV, PRRSV, SIV and ADV

PCV2 was detected by real time qPCR according to previous protocols by Cadar et al. [35]; TTSuV1–2 by classical one step PCR according to previous protocols by Segales et al. [36]; PPV1 was checked using nested PCR according to Soares et al. [37]; PPV2–4, PoBoV1–4, PoBolV was detected by classical PCR according to previous protocols by Zhai et al., Csagola et al. and Ndze et al. [15, 38, 39]; PCMV was detected by classic PCR according to previous protocol by Hamel et al. [40]; 6 V/7 V by classical PCR according previous protocol by Kiss et al. [41]; ADV was detected by real-time PCR according to previous protocol by McKillen et al. [42]; PRRSV and SIV were detected by real time reverse nested RT PCR according to previous protocol by Biernacka et al. [43].

### Immunohistochemistry to detect CD3, SLAIIDQ, lysozyme, PRRSV, SIV and *Mycoplasma hyopneumoniae*

Lung sections were dewaxed with xylene and rehydrated through graded alcohols to water. Endogenous peroxidase activity was blocked by incubation with 3% hydrogen peroxide in 0.1 M Tris-buffered saline (TBS, pH = 7.6) for 30 min. Immunohistochemistry was performed with seven primary antibodies: anti-human CD3, anti-SLAIIDQ, anti-lysozyme anti-PRRSV, anti-H1N1/H3N2 and anti-Mhyo with specific pretreatment and dilutions given in Additional file 11 as described previously [44–46]. For visualization of the positive reactions an anti-rabbit and anti-mouse labelled polymer HRP conjugated detection system (Envision, Dako) was used with a final incubation in diaminobenzidine (DAB)–hydrogen peroxide solution for 5 min (Dako, Denmark). Slides were counterstained in Mayer’s haematoxylin, dehydrated, covered-slipped and microscopically examined. Lymph node from a slaughtered healthy pig served as positive control tissue. As negative controls, irrelevant primary antibodies at the same dilution were used as substitutes for the specific antibodies. The immune response observed in the lungs was graded as no immune cells or “0”, weak infiltration or “1”, moderate infiltration or “2” and severe infiltration or “3”. For positive grading controls, lung sections positive for PRRSV, SIV or *Mhyo* were used. Negative controls were prepared by omitting the primary antibodies.

### In situ hybridization to detect PCV2

To detect PCV2 DNA in tissues, ISH was carried out on paraffin-embedded samples as previously described [47]. The level of PCV2 ssDNA and/or dsDNA was estimated in different cell types.

### In situ polymerase chain reaction (IS-PCR)

Direct IS-PCR was performed as previously described [23, 24, 48–50], with some modifications. Sections were dewaxed in xylene and rehydrated through different grades of ethanol to water. Dewaxed sections on DNase/RNase-free electrocharged slides were incubated with 40 μl Proteinase K (Dako) in 2 ml TBS (according to manufacturer’s instructions) for 3 min at room temperature. Direct in situ PCR was performed using specific primers forward PPV2AF 5^’^-ACACGATGAGCGGTACGA-3′ and reverse PPV2AR 5^’^-TCCTCACGAGGTCTCTTCTG-3^′^ to amplify a 279 bp long ORF2 region sequence (GenBank access number NC_035180) as previously described [15]. After thorough washing of the slides with DEPC-treated water, 100% ethanol and air dry, 10 μl of PCR optimal solution (master mix) containing digoxigenin-11-(2′-deoxy-uridine-5′)-triphosphate (DIG-11-dUTP; Roche, USA) was added [23] on dried cut and the slides were covered. For amplification a Hot Start polymerase (Qiagen) was used. Cycling conditions and primers (Sigma-Aldrich) were used as described [15] and were further optimized for IS-PCR, such as increasing the denaturing and annealing temperatures by 1 °C and expanding to 15 s [24, 48–50]: activation step 95 °C/5 min; denaturation 95 °C/45 s, elongation 72 °C/1 min, annealing 57 °C/55 s and final elongation 72 °C/5 min during 30 cycles in in situ PCR thermal cycler (Perkin Elmer Geneamp In situ PCR System 1000 Thermal Cycler). Slides were stored overnight at 4 °C to immobilize in situ the amplicons. Sections were then washed 3X in 0.5 and 3X in 0.25 Saline-Sodium Citrate (SSC) buffer (Sigma-Aldrich) and finally 3X in Tris buffer pH 7.6. The detection was performed as described for ISH. To exclude primer independent amplification, positive samples were repeatedly tested with either the forward or reverse primer. As a negative control a paraffin block tissue from an animal that was PCR negative for PPV2 was used.

### Double staining in situ PCR/IHC

Double staining to detect PPV2 and B and T lymphocytes and monocytes/macrophages was performed using a previously described protocol for in situ PCR to detect PPV2 and IHC to detect host markers CD3, SLAIIDQ and lysozyme. The forward order was detection of PPV2 nucleic acid first and then detection of host antigens (CD3, SLAIIDQ and lysozyme), while the reverse order started with the detection of the host antigens (CD3, SLAIIDQ and lysozyme) and then the viral nucleic acid.

### IHC-is-PCR

Slides were processed with IS-PCR protocol until and incubated with NBT/BCIP to visualize positive signal for PPV2 presence without counterstaining. After incubation, the slides were placed in TBS and the appearance of blue chromogen was monitored under light microscope and were routinely subject to IHC protocol. Tissues were not pre-treated with *pronase* for CD3 and Lysozime detection but were incubated in citrate buffer pH 6 for 20 min at 96 °C for SLAIIDQ detection. Endogenous peroxidase activity was blocked by incubation with 3% hydrogen peroxide in TBS for 30 min. Tissue sections were rinsed in TBS and incubated with 20% normal goat serum solution in TBS for 1 h at room temperature.

### Is-PCR-IHC

The slides were routinely subjected to IHC to detect CD3, SLAIIDQ and lysozyme followed by an incubation with DAB to visualize positive reaction. After incubation with DAB solution, without counterstaining, slides were placed in 100% ethanol and the appearance of brown chromogen was monitored under light microscope. Slides were then routinely processed to IS-PCR as previously described. Controls for IHC or in situ PCR were performed in single reaction.

### Statistical analysis

Frequencies of pathological lesions and severity of immune cell infiltrations were assessed. The studied groups of samples were PCR positive and negative for the presence of PPV2; PMWS affected and non-affected (PCV2 positive) and PCV2-negative animals. All statistical analyses were performed using Statistical Analysis System (SAS®) for windows 6.14 (SAS Institute Inc., Cary, NC, USA). Depending on the parameterization (coding) of categorical explanatory variable we used GENMOD or LOGISTIC procedures and as our sample size was small we never used more than two explanatory variables in the model neither their interactions. Thus, the effects of age (defined in months, covariate in models) and/or other relevant dependent variables (see Additional files 5, 6 and 7), on the prevalence of PPV2 (categorized as 0 or 1) were analyzed by univariate generalized linear models (PROC GENMOD with normal distribution and link function identity). Furthermore, the effects of relevant dependent variables (see Additional file 2) on the prevalence of PMWS (categorized as 0 or 1) were analyzed by logistic regression (PROC LOGISTIC with link function logit and option ODDSRATIO).

## Additional files

Additional file 1: Table presents results of PCR test for presence of following viruses: TTSuV, PPV1–4, PoBoV1–4, PoBolV, 6 V/7 V, PCMV, PRRSV, SIV and ADV in farm E. (XLS 30 kb)

Additional file 2: ODS results Farm E– PPV2 vs viruses. (DOCX 14 kb)

Additional file 3: Table presents results of histopathological examination of lungs in farm E. (XLS 27 kb)

Additional file 4: Table presents results of presence of PCV2 in lungs tested by ISH in farm E. (XLS 33 kb)

Additional file 5: Wald Chi2-square Farm E: Histopathology. (DOCX 15 kb)

Additional file 6: Wald Chi2-square Farm E: ISH PCV2. (DOCX 16 kb)

Additional file 7: Wald Chi2-square Farm E: IHC immune cells. (DOCX 16 kb)

Additional file 8: Results of IHC to detect specific immune cell markers in farm E. (XLS 22 kb)

Additional file 9: Presence of PPV2 by in situ PCR, histopathology findings and immune cell reaction in farm T. (XLSX 11 kb)

Additional file 10: Presence of PPV2 by in situ PCR and other pathogens in farm T. (XLSX 9 kb)

Additional file 11: Details of primary antibodies used for IHC. (DOCX 23 kb)

## Abbreviations

6 V/7 V: Porcine adenovirus
ADV: Aujezsky disease virus
B&B: Brown and Brenn
IHC: Immunohistochemistry
IS PCR: In situ polymerase chain reaction
*Mhyo*: *Mycoplasma hyopneumoniae*
ORFs: Open reading frames
PCMV: Porcine cytomegalovirus
PCV2: Porcine circovirus type 2
PMWS: post-weaning multi-systemic wasting syndrome
PoBolV: Porcine boca like virus
PoBoV: Porcine bocavirus
PPV: Porcine parvovirus
PRRSV: Porcine reproductive and respiratory syndrome virus
SIV: Swine influenza
SLAIIDQ: Swine leukocyte antigen class II DQ
TBS: Tris-buffered saline
TTSuV: Torque teno sus virus
