# Supplementary material for: Investigating porcine parvoviruses genogroup 2 infection using in situ polymerase chain reaction
Source: BMC Vet Res. 2018 May 21;14:163. doi: 10.1186/s12917-018-1487-z (PMC5963090; doi:10.1186/s12917-018-1487-z)
Supplement: Supplementary file 2 — ODS results Farm E– PPV2 vs viruses. (DOCX 14 kb) [file 12917_2018_1487_MOESM2_ESM.docx]

**Additional file 2: SAS: ODS results Farm E– PPV2 vs viruses**

|  | DF | Estimate | StdErr | Wald Chi-Square | Pr>ChiSq | Point Estimate | 95% Wald Confidence Limits | |
| --- | --- | --- | --- | --- | --- | --- | --- | --- |
| PPV2 | 1 | 1.1192 | 0,7657 | 2.1365 | 0.1438 | 3.062 | 0.683 | 13.735 |
| PPV3 | 1 | -12.368 | 0.7846 | 24.847 | 0.1150 | 0.290 | 0.062 | 1.351 |
| PPV4 | 1 | 0.3407 | 11.585 | 0.0865 | 0.7687 | 1.406 | 0.145 | 13.618 |
| PoBolV | 1 | 0.2719 | 0.7142 | 0.1450 | 0.7034 | 1.313 | 0.324 | >999.999 |
| PoBoV3 | 1 | 114.422 | 403.8 | 0.0008 | 0.9774 | >999.999 | <0.001 | >999.999 |
| SIV | 1 | 0.3407 | 11.585 | 0.0865 | 0.7687 | 1.406 | 0.145 | 13.618 |
| TTSuV1 | 1 | 0.4549 | 0.7389 | 0.3789 | 0.5382 | 1.576 | 0.307 | 6.707 |
| TTSuV2 | 1 | -0.4965 | 0.7176 | 0.4788 | 0.4890 | 0.609 | 0.149 | 2.484 |
| PRRSV | 1 | -15.805 | 0.9159 | 29.774 | 0.0844 | 0.206 | 0.034 | 1.240 |
