# Supplementary material for: Investigating porcine parvoviruses genogroup 2 infection using in situ polymerase chain reaction
Source: BMC Vet Res. 2018 May 21;14:163. doi: 10.1186/s12917-018-1487-z (PMC5963090; doi:10.1186/s12917-018-1487-z)
Supplement: Supplementary file 5 — Wald Chi2-square Farm E: Histopathology. (DOCX 15 kb) [file 12917_2018_1487_MOESM5_ESM.docx]

**Additional file 5: SAS: Wald Chi2 Farm E: Histopathology**

| **Value^*^** | **Parameter** | **DF** | **Estimate** | **Standard Error** | **Wald 95% Confidence Limits** | | **Wald Chi-Square** | **Pr > ChiSq** |
| --- | --- | --- | --- | --- | --- | --- | --- | --- |
| alv_wal_pro | 0 | 1 | 0.1404 | 0.3080 | -0.4633 | 0.7440 | 0.21 | 0.6486 |
|  | 1 | 1 | 0.2051 | 0.3175 | -0.4172 | 0.8275 | 0.42 | 0.5183 |
|  | 2 | 1 | 0.2500 | 0.3200 | -0.3772 | 0.8772 | 0.61 | 0.4347 |
| alv_spa_red | 0 | 1 | -0.1758 | 0.1755 | -0.5197 | 0.1681 | 1.00 | 0.3163 |
|  | 1 | 1 | 0.0385 | 0.2047 | -0.3628 | 0.4397 | 0.04 | 0.8510 |
|  | 2 | 1 | 0.3718 | 0.1824 | 0.0143 | 0.7292 | **4.16** | **0.0415** |
| alv_spa_inf_sei | 0 | 1 | -0.4000 | 0.1832 | -0.7591 | -0.0409 | **4.77** | **0.0290** |
|  | 1 | 1 | -0.3167 | 0.1832 | -0.6757 | 0.0424 | **2.99** | **0.0839** |
|  | 2 | 1 | -0.2333 | 0.2071 | -0.6392 | 0.1726 | **1.27** | **0.2599** |
| ilb_ede | 0 | 1 | 0.1333 | 0.2398 | -0.3366 | 0.6033 | 0.31 | 0.5782 |
|  | 1 | 1 | 0.1714 | 0.2907 | -0.3982 | 0.7411 | 0.35 | 0.5553 |
|  | 2 | 1 | 0.0000 | 0.3139 | -0.6153 | 0.6153 | 0.00 | 10.000 |
| con | 0 | 1 | 0.1310 | 0.1833 | -0.2284 | 0.4903 | 0.51 | 0.4750 |
|  | 1 | 1 | -0.3167 | 0.1805 | -0.6704 | 0.0371 | 3.08 | 0.0793 |
|  | 2 | 1 | -0.0833 | 0.2330 | -0.5400 | 0.3734 | 0.13 | 0.7206 |

Abbreviation: * alv_wal_pro – proliferation of alveolar walls; alv_spa_red – reduction of space in alveolas; alv_spa_inf_sei – seize of infiltration in alveolas; ilb_ede – interlobar edema; con – lung congestion
