# Supplementary material for: Investigating porcine parvoviruses genogroup 2 infection using in situ polymerase chain reaction
Source: BMC Vet Res. 2018 May 21;14:163. doi: 10.1186/s12917-018-1487-z (PMC5963090; doi:10.1186/s12917-018-1487-z)
Supplement: Supplementary file 6 — Wald Chi2-square Farm E: ISH PCV2. (DOCX 16 kb) [file 12917_2018_1487_MOESM6_ESM.docx]

**Additional file 6: SAS: Wald Chi2 Farm E: ISH PCV2**

| **Value ^1, 2^** | **Parametar** | **DF** | **Estimate** | **Standard Error** | **Wald 95% Confidence Limits** | | **Wald Chi-Square** | **Pr > ChiSq** |
| --- | --- | --- | --- | --- | --- | --- | --- | --- |
| CP_mac_alv_spa | 0 | 1 | 0.1261 | 0.2121 | -0.2896 | 0.5417 | 0.35 | 0.5522 |
|  | 1 | 1 | 0.4476 | 0.2161 | 0.0240 | 0.8712 | **4.29** | **0.0384** |
|  | 2 | 1 | 0.2143 | 0.2444 | -0.2647 | 0.6933 | 0.77 | 0.3806 |
| CP_cel_alv_wal | 0 | 1 | 0.4000 | 0.3097 | -0.2069 | 10.069 | 1.67 | 0.1964 |
|  | 1 | 1 | 0.6667 | 0.2933 | 0.0917 | 12.416 | **5.16** | **0.0230** |
|  | 2 | 1 | 0.5000 | 0.2960 | -0.0801 | 10.801 | 2.85 | 0.0911 |
| CP_pbr_cel | 0 | 1 | 0.0000 | 0.2000 | -0.3921 | 0.3921 | 0.00 | 10.000 |
|  | 1 | 1 | 0.2500 | 0.2450 | -0.2302 | 0.7302 | 1.04 | 0.3075 |
|  | 2 | 1 | 0.1716 | 0.1847 | -0.1905 | 0.5337 | 0.86 | 0.3531 |
| CP_cil_epi_cel | 0 | 1 | 0.0833 | 0.3186 | -0.5412 | 0.7079 | 0.07 | 0.7937 |
|  | 1 | 1 | 0.2381 | 0.3047 | -0.3591 | 0.8353 | 0.61 | 0.4345 |
|  | 2 | 1 | 0.2121 | 0.3215 | -0.4181 | 0.8423 | 0.44 | 0.5094 |
| CP_blo_wes | 0 | 1 | -0.0627 | 0.1515 | -0.3596 | 0.2341 | 0.17 | 0.6787 |
| CP_mac | 0 | 1 | -0.3476 | 0.2310 | -0.8003 | 0.1051 | 2.26 | 0.1323 |
| CP_pne | 0 | 1 | -0.2805 | 0.1578 | -0.5898 | 0.0287 | 3.16 | 0.0754 |
| CP_epi_cel | 0 | 1 | -0.1742 | 0.1610 | -0.4898 | 0.1414 | 1.17 | 0.2793 |
| CP_end | 0 | 1 | 0.1109 | 0.1530 | -0.1890 | 0.4108 | 0.53 | 0.4687 |
| CP_lym | 0 | 1 | -0.5111 | 0.3535 | -12.039 | 0.1817 | 2.09 | 0.1482 |
| RFP_mac_alv_spa | 0 | 1 | 0.2652 | 0.2607 | -0.2457 | 0.7760 | 1.03 | 0.3090 |
|  | 1 | 1 | 0.3500 | 0.2913 | -0.2209 | 0.9209 | 1.44 | 0.2295 |
| RFP_cel_alv_wal | 0 | 1 | 0.5000 | 0.2055 | 0.0972 | 0.9028 | **5.92** | **0.0150** |
|  | 1 | 1 | 0.7059 | 0.2138 | 0.2868 | 11.250 | **10.90** | **0.0010** |
| RFP_cil_epi_cel | 0 | 1 | -0.1667 | 0.3001 | -0.7548 | 0.4215 | 0.31 | 0.5786 |
|  | 1 | 1 | -0.1667 | 0.3280 | -0.8095 | 0.4761 | 0.26 | 0.6113 |
| RFP_blo_wes | 0 | 1 | 0.2033 | 0.2165 | -0.2210 | 0.6275 | 0.88 | 0.3478 |
| RFP_mac | 0 | 1 | -0.1216 | 0.1507 | -0.4170 | 0.1738 | 0.65 | 0.4199 |
| RFP_pne | 0 | 1 | 0.0152 | 0.1594 | -0.2973 | 0.3276 | 0.01 | 0.9243 |
| RFP_end | 0 | 1 | 0.0111 | 0.3612 | -0.6969 | 0.7191 | 0.00 | 0.9755 |
| RFP_lym | 0 | 1 | 0.0111 | 0.3612 | -0.6969 | 0.7191 | 0.00 | 0.9755 |

Abbreviation: ^1^ CP – ISH was performed using Complementary genomic probe; RFP – ISH was performed using Replicative form genomic probe. ^2^ mac_alv_spa – macrophages in alveolar spaces; cel_alv_wal – cells in alveolar walls; cil_epi_cel – cilliar epithelial cells in bronchi; mac - macrophages; pne - pneumocytes; end – endothelial cells; lym – lymphocytes.
