# Supplementary material for: Investigating porcine parvoviruses genogroup 2 infection using in situ polymerase chain reaction
Source: BMC Vet Res. 2018 May 21;14:163. doi: 10.1186/s12917-018-1487-z (PMC5963090; doi:10.1186/s12917-018-1487-z)
Supplement: Supplementary file 7 — Wald Chi2-square Farm E: IHC immune cells. (DOCX 16 kb) [file 12917_2018_1487_MOESM7_ESM.docx]

**Additional file 7: SAS: Wald Chi2 Farm E: IHC immune cells**

| **Value^1, 2^** | **Parameter** | **DF** | **Estimate** | **Standard Error** | **Wald 95% Confidence Limits** | | **Wald Chi Square** | **Pr > ChiSq** |
| --- | --- | --- | --- | --- | --- | --- | --- | --- |
| **lu_CD3** | **PPV2** | **1** | **-19.167** | **0.8513** | **-35.851** | **-0.2482** | **5.07** | **0.0244** |
|  | **PCV2** | **1** | **0.0167** | **0.7020** | **-13.592** | **13.925** | **0.00** | **0.9811** |
| **lu_DQ** | **PPV2** | **1** | **-0.5000** | **0.9825** | **-24.256** | **14.256** | **0.26** | **0.6108** |
|  | **PCV2** | **1** | **-0.1667** | **0.8102** | **-17.546** | **14.213** | **0.04** | **0.8370** |
| **lu_Ly** | **PPV2** | **1** | **-0.5833** | **10.164** | **-25.754** | **14.087** | **0.33** | **0.5660** |
|  | **PCV2** | **1** | **-29.167** | **0.8381** | **-45.593** | **-12.740** | **12.11** | **0.0005** |
| **lu_sigma** | **PPV2** | **1** | **-30.000** | **18.514** | **-66.287** | **0.6287** | **2.63** | **0.1052** |
|  | **PCV2** | **1** | **-30.667** | **15.267** | **-60.590** | **-0.0743** | **4.03** | **0.0446** |
| **lu_wal_CD3** | **PPV2** | **1** | **-15.000** | **0.4640** | **-24.094** | **-0.5906** | **10.45** | **0.0012** |
|  | **PCV2** | **1** | **-0.8333** | **0.3826** | **-15.832** | **-0.0834** | **4.74** | **0.0294** |
| **lu_spa_CD3** | **PPV2** | **1** | **0.1667** | **0.3184** | **-0.4574** | **0.7908** | **0.27** | **0.6007** |
|  | **PCV2** | **1** | **-0.1000** | **0.2626** | **-0.6146** | **0.4146** | **0.15** | **0.7033** |
| **lu_pbr_CD3** | **PPV2** | **1** | **-15.833** | **0.5346** | **-26.311** | **-0.5356** | **8.77** | **0.0031** |
|  | **PCV2** | **1** | **-0.3833** | **0.4408** | **-12.473** | **0.4807** | **0.76** | **0.3845** |
| **lu_pva_CD3** | **PPV2** | **1** | **-0.3333** | **0.4441** | **-12.037** | **0.5371** | **0.56** | **0.4529** |
|  | **PCV2** | **1** | **0.4667** | **0.3662** | **-0.2511** | **11.844** | **1.62** | **0.2026** |
| **lu_wal_DQ** | **PPV2** | **1** | **-0.1667** | **0.3184** | **-0.7908** | **0.4574** | **0.27** | **0.6007** |
|  | **PCV2** | **1** | **0.2333** | **0.2626** | **-0.2813** | **0.7480** | **0.79** | **0.3742** |
| **lu_spa_DQ** | **PPV2** | **1** | **0.0833** | **0.2783** | **-0.4621** | **0.6287** | **0.09** | **0.7646** |
|  | **PCV2** | **1** | **-0.0500** | **0.2295** | **-0.4997** | **0.3997** | **0.05** | **0.8275** |
| **lu_pbr_DQ** | **PPV2** | **1** | **-0.1667** | **0.4362** | **-10.216** | **0.6883** | **0.15** | **0.7024** |
|  | **PCV2** | **1** | **-0.0333** | **0.3597** | **-0.7383** | **0.6717** | **0.01** | **0.9262** |
| **lu_pva_DQ** | **PPV2** | **1** | **-0.2500** | **0.3759** | **-0.9868** | **0.4868** | **0.44** | **0.5060** |
|  | **PCV2** | **1** | **-0.3167** | **0.3100** | **-0.9242** | **0.2909** | **1.04** | **0.3070** |
| **lu_wal_Ly** | **PPV2** | **1** | **-0.0833** | **0.4179** | **-0.9024** | **0.7358** | **0.04** | **0.8419** |
|  | **PCV2** | **1** | **-0.2167** | **0.3446** | **-0.8921** | **0.4588** | **0.40** | **0.5295** |
| **lu_spa_Ly** | **PPV2** | **1** | **-0.2500** | **0.5293** | **-12.875** | **0.7875** | **0.22** | **0.6367** |
|  | **PCV2** | **1** | **-0.6500** | **0.4365** | **-15.055** | **0.2055** | **2.22** | **0.1365** |
| **lu_pbr_Ly** | **PPV2** | **1** | **-0.4167** | **0.5187** | **-14.334** | **0.6001** | **0.65** | **0.4218** |
|  | **PCV2** | **1** | **-14.167** | **0.4278** | **-22.551** | **-0.5783** | **10.97** | **0.0009** |
| **lu_pva_Ly** | **PPV2** | **1** | **0.1667** | **0.4200** | **-0.6565** | **0.9898** | **0.16** | **0.6915** |
|  | **PCV2** | **1** | **-0.6333** | **0.3463** | **-13.121** | **0.0455** | **3.34** | **0.0674** |

Abbreviation: ^1^ lu- lungs; wal – alveolar walls; spa -alveolar spaces; pbr – peribronchial area; pva – perivascular area. ^2^ CD3 – IHC was performed using anti-CD3 Mab; IHC was performed using anti-SLAIIDQ Mab; IHC was performed using anti-Lysozyme Mab, sigma – means all observed inflammatory cells.
