# Supplementary material for: Investigating porcine parvoviruses genogroup 2 infection using in situ polymerase chain reaction
Source: BMC Vet Res. 2018 May 21;14:163. doi: 10.1186/s12917-018-1487-z (PMC5963090; doi:10.1186/s12917-018-1487-z)
Supplement: Supplementary file 11 — Details of primary antibodies used for IHC. (DOCX 23 kb) [file 12917_2018_1487_MOESM11_ESM.docx]

Additional file 8.

Details of primary antibodies used for IHC.

| Specificity | pAb/mAb (clone) | Host of origin | Type | Treatment | Dilution | Source |
| --- | --- | --- | --- | --- | --- | --- |
| CD3 | pAb  (DAKO | Human | Rabbit polyclonal | *Pronase^a^* | 1/100 | Dako (Denmark) |
| SLAIIDQ | mAb (MCA1335) | Swine | Mouse monoclonal | *Pronase^a^* | 1/200 | AbD Serotec  (UK) |
| Lysozyme | pAb (A099) | Human | Rabbit polyclonal | *Pronase^a^* | 1/500 | Dako (Denmark) |
| PRRSV | mAb  (1AC7) | NC protein | Mouse monoclonal | *Pronase^c^* | 1/200 | Ingenasa (Spain) |
| SIV (H1N1/H3N2) | mAb  (3DH6) |  | Mouse monoclonal | *Pronase^c^* | 1/200 | Ingenasa (Spain) |
| *Mycoplasma hyoppneumoniae* | mAb  (8B.I3) | OMP antigen | Mouse monoclonal | *Heating^b^* | 1/500 | JenoBiotech  (Korea) |

^a^Incubation with Proteinase K for 3 min at room temperature

^b^Incubation in citrate buffer pH6 for 20 min at 96°C

^c^Incubation with Protease 14 for 10 min at 37°C
